# Monitoring and Evaluating Progress towards Universal Health Coverage in Estonia

**DOI:** 10.1371/journal.pmed.1001677

**Published:** 2014-09-22

**Authors:** Taavi Lai, Triin Habicht, Maris Jesse

**Affiliations:** 1 Department of Public Health, University of Tartu, Estonia; 2 Department of Health Care, Estonian Health Insurance Fund, Estonia; 3 National Institute for Health Development, Estonia

## Abstract

This paper is a country case study for the Universal Health Coverage Collection, organized by WHO. Taavi Lai and colleagues illustrate progress towards UHC and its monitoring and evaluation in Estonia.

*Please see later in the article for the Editors' Summary*

This paper is part of the PLOS Universal Health Coverage Collection. This is the summary of the Estonia country case study. The full paper is available as Supporting Information file Text S1.

## Background

Since regaining its independence in 1991, Estonia has conducted radical health system reforms. The Estonian health system is based on mandatory, solidarity-based insurance and universal access to health services made available by providers operating under private law with primary health care (PHC) playing a central role. The financing of health care is mainly organized through the semi-autonomous Estonian Health Insurance Fund (EHIF), which covers about 70% of total health expenditure in the country. Life expectancy at birth (LE) among a population of 1.3 million reached 76.2 years in 2012.

## Universal Health Coverage: The Policy Context

Health policy issues are mostly covered at the national level, while municipalities have only a limited role in public health policy through local Health in All Policies (inclusion of health considerations in policy making across different sectors that influence health) and implementation of selected national public health programmes. Currently, the main policy document is the National Health Plan (NHP) 2009–2020 [1], which contains a wide set of measurable targets with specific indicators that are reported annually with outcome reviews every second year.

## Monitoring and Evaluation for UHC

While the NHP was adopted as an overarching health policy only in 2008, there is a longer tradition in Estonia regarding area-specific health strategies [2]–[4] and institutional development plans [5]. These strategies and plans all have specialised indicator frameworks for targeting, monitoring, and assessing progress made in improving population health. All these specific policy documents feed into the overall health system monitoring and assessment system, thus enabling improved governance and policy development.

The monitoring and reporting system of the NHP is further supported by the health system performance assessment (HSPA) [6], explicitly linked to the health system framework that prioritises health outcomes, financial protection, and responsiveness to need, which are also the main principles of UHC.

A UHC-specific monitoring framework is not present in Estonia but its main components are present in the NHP and HSPA indicator frameworks.

## Progress towards UHC in Estonia

### Financial Protection

**Figure 1:**
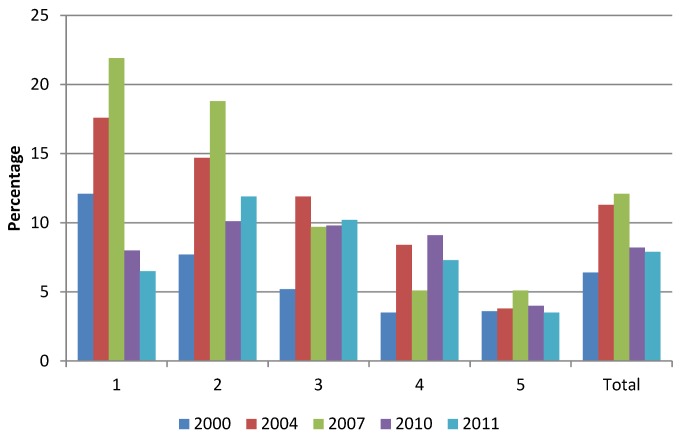
Out-of-pocket payments as a proportion of total household expenditure by quintiles [8].

In Estonia, population coverage with health insurance is 95%, and the entire population is covered for emergency care. Share of out-of-pocket (OOP) payments declined from 25% in 2006 to 18% in 2012 (Figure 1) [7],[8]; the remainder was covered by public funds (mainly health insurance). The main source of OOP payments is co-payments for outpatient medication and adult dental care.

Household budget data show that in 2011 the proportion of high health expenditures (>20% of capacity to pay) was lowest among the poorest and richest income quintiles with 6.5% and 3.5%, respectively, while in the second income quintile this proportion reached 12%. As a result of OOP payments, about 1% of households in the poorest quintile faced poverty in 2011 compared to almost 9% in 2004.

### Responsiveness to Need

Access to health care services was considered to be good or very good in Estonia by 55% of the adult population and 79% was satisfied with the quality of care in 2012 [9].

Coverage with services is monitored using utilization data. Service coverage is further supported by a PHC quality bonus system [10], which includes indicators for monitoring disease prevention, chronic disease management, and additional activities, as surgical and gynaecological procedures at the PHC level use annually increasing targets for all indicators (45 in 2013).

Efficiency of service provision is important for identifying possibilities for extending UHC with available resources. The volume of acute care hospital beds has decreased significantly over the past 20 years and by 2011 reached 350 per 100,000 people (below the European Union [EU] average) [11]. The average length of these acute care stays decreased from 17.4 days in 1990 to 5.5 days in 2011. The number of physicians has remained stable (326 per 100,000 in 2011) with a nurse to physician ratio of two (approximately three in the EU) since the 1990s.

Service quality measurement is one of the areas in development in Estonia with a first set of health care quality indicators for acute care hospitals published in 2011 [12]. In parallel, 92% of people were satisfied with their last visit to a family doctor, 93% with their last hospital visit, and 88% with their last outpatient specialist visit in 2012 [9].

### Health Outcomes

**Figure 2:**
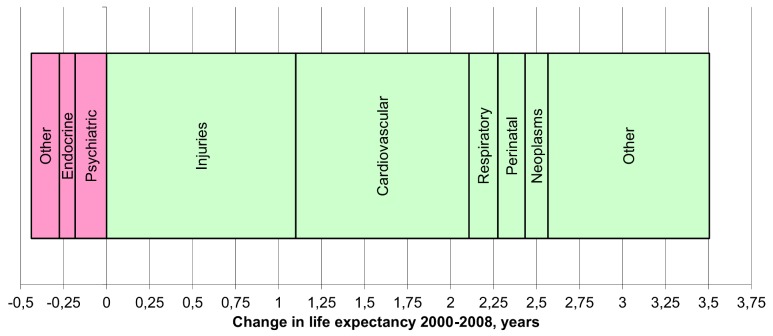
Life expectancy change in Estonia between 2000 and 2008 by cause of death [6].

LE has increased by about 10 years from its lowest in 1994 to 76.2 years in 2012 [13]. However, a ten-year gender gap favouring women has persisted since 1991. The LE increase has been driven by reductions in cardiovascular, injury, and cancer mortality (accounting for 93% of LE improvement during 2000–2008), whereas diabetes and alcohol-related conditions slowed the LE increase (Figure 2) [6].

Health inequalities in Estonia are mainly related to socio-economic factors; the proportion of individuals assessing their health as good or very good is persistently 20 or more percentage points higher among the highly educated and high-income groups compared to those with the lowest education levels [10],[14].

## Conclusions and Recommendations

Estonia has been successful in achieving UHC although work remains to reach absolute universal coverage, such as in extending health insurance coverage, reducing the share of OOP payments, and addressing health inequalities.

The Estonian experience has shown that comprehensive policy monitoring and assessment enables the monitoring of UHC even in the absence of a dedicated framework. In particular, UHC monitoring can be facilitated if extensive routine data sources are developed, linked, and integrated through comprehensive IT solutions. However, a specific focus on UHC and creating a dedicated monitoring framework within the existing system would target existing data gaps and move UHC into strong policy focus for systematic policy development.

## Supporting Information

Text S1 **The full country case study for Estonia.**
(DOCX)

## Abbreviations

LE: life expectancy
NHP: National Health Plan
OOP: out-of-pocket
PHC: primary health care
