# Supplementary material for: Monitoring and Evaluating Progress towards Universal Health Coverage in Estonia
Source: PLoS Med. 2014 Sep 22;11(9):e1001677. doi: 10.1371/journal.pmed.1001677 (PMC4170953; doi:10.1371/journal.pmed.1001677)
Supplement: Text S1 — The full country case study for Estonia. (DOCX) [file pmed.1001677.s001.docx]

**Full Case Study: Monitoring and evaluating progress towards Universal Health Coverage in Estonia**

Taavi Lai^1^, Triin Habicht^2^, Maris Jesse^3^

^1^ Department of Public Health, University of Tartu, Estonia

^2^ Department of Health Care, Estonian Health Insurance Fund, Estonia

^3^ National Institute for Health Development, Estonia

*Corresponding author: Taavi Lai

Email: taavi.lai@gmail.com

**This paper is the full country case study to accompany the summary paper “Monitoring and evaluating progress towards Universal Health Coverage in Estonia” that is part of the Universal Health Coverage Collection. Not commissioned; externally reviewed.**

**Abstract:** Universal health coverage (UHC) is defined as a situation where all people who need health services receive them without incurring financial hardship and this concept as a goal for health policy development has gained wide acceptance in the world since the publication of the World Health Report 2010. The purpose of this paper is to provide an example of country level UHC measurement based on the current policy monitoring and assessment system in Estonia.

The components of UHC can all be found in existing Estonian policy documents, health system performance reporting and their respective monitoring systems. While there is no separate policy, UHC can be monitored and assessed within the existing policy development framework present in Estonia. Indicators for monitoring UHC presented in this paper are divided into three dimensions: financial protection, coverage with services and health outcomes. Using variety of indicators in these three dimensions, Estonian situation of UHC is described.

We find that specific focus on UHC in the existing policy framework would be one of the key enablers to take progress toward UHC forward as there are several possibilities to further elaborate and extend existing monitoring and data collection systems for evidence based policy development on UHC. Among these possibilities, more systematic use of existing possibilities of the excellent health information system, introduction of population health examination surveys in integration with existing data sources and specific focus on development and use of equity sensitive indicators stand out.

**Summary Points:**

1. Comprehensive and systematic policy development and monitoring framework present in Estonia with growing performance assessment tradition

2. Constituent parts of UHC present in existing policy and performance assessment framework but UHC as a comprehensive approach not clearly defined there

3. Estonian experience shows that comprehensive policy monitoring and assessment system with underlying health system framework enables monitoring of UHC even without a dedicated UHC monitoring framework.

4. Specific focus on UHC within the existing policy and assessment framework would help to progress toward UHC

5. Health information systems in Estonia are very well developed and form an excellent base for further development of UHC monitoring and assessment system

6. There are several possibilities to extend existing monitoring frameworks for improved UHC measurement especially in the equity dimension and areas of service quality and effective coverage

**1. Background**

The concept of universal health coverage (UHC) as a goal of health policy development has gained wide acceptance at country and global levels since the publication of the World Health Report 2010 [1]. UHC has been defined as a situation where all people who need health services receive them, without incurring financial hardship [1] and thus consists of two components: coverage with needed quality health services and access to financial risk protection, for everyone. The former captures the aspiration that all people obtain the health services they need, while the latter aims to ensure that they do not suffer financial hardship linked to paying for these services. The level and distribution of (effective) coverage of interventions and financial risk protection have been proposed as the focus of monitoring progress towards UHC [2]. Universal health coverage is not about a minimum service package or a one-size-fits-all approach to service delivery and financial risk protection, but rather emphasizes progressive realization of coverage according to a country’s situation. Ultimately, improved UHC should also be reflected in the main goal of all health systems – improved population health both in terms of absolute levels and distribution of the health in population groups.

The purpose of this paper is to provide an example of country level UHC measurement system. Firstly, we provide an overview of the health policy monitoring and assessment system in Estonia. Secondly, we present examples of UHC measurement based on the existing indicator framework of the National Health Plan 2009-2020 [3] and health system performance assessment reporting [4]. Finally, we summarise best practices as well as main barriers and enablers for systematic measurement of UHC based on our country-experience.

Country situation

Estonia is a small country in the North-Eastern Europe and member of European Union since 2004 and of Euro currency zone since 2011. After regaining independence from Soviet Union in 1991 radical and comprehensive reforms were pursued in different sectors including health. Mandatory social health insurance, reduced and restructured hospital network and primary health care centred system were the principal outcomes of health sector reforms. The solidarity-based health insurance provides universal access to health services made available by providers operating under private law. Health insurance covers about 70% of total health care expenditure in Estonia with total public funding above 80% and about 18% remaining to out-of-pocket (OOP) payments [5].

In parallel to other reforms, electronic registries and databases containing information on every health, welfare and mortality event in the country were created [6] using unique national personal identifier (ID). Latter enables linkage of all data sources in health and across sectors with technical possibility to track one individual over data sources and time if data protection criteria are fulfilled.

Estonian population of 1.3 million is ageing and declining while average life expectancy (LE) has increased since 1990 reaching 76.2 years in 2012 [7] with male LE constantly about 10 years lower compared to female. Infant mortality had a 3-fold reduction since 1992, down to 2.4 infant deaths per 1000 live births in 2011 [8]. These rapid changes can all be attributed to the cardinal reforms transforming Estonia from a post-soviet to a modern European country both society and health system wise [6].

However, health behaviour in Estonia is of concern as 10.4 litres of pure alcohol was consumed per capita [9], 26% of adults smoked daily and 19% were obese in 2012 [10]. This is directly linked to the main health problems in Estonia – cardiovascular diseases, cancers and injuries while musculoskeletal diseases and mental health problems are gaining importance as well [11,12].

The economic reforms have enabled Estonia to become high-income country with average annual growth of GDP almost 9% in real terms before economic crisis [13]. However, distribution of wealth in Estonia is slightly less equal than the EU average. Still, Gini coefficient has declined from 37.4 in 2004 to 31.9 in 2011 (30.7 in EU on average in 2011) [14]. The proportion of people in poverty or at risk of social exclusion was 23.1% in Estonian population compared to 24.2% in the EU in 2011 [14]. The trend here is also positive as only in 2005 people in poverty constituted 25.9% compared to EU average 25.6%. Thus, the overall outlook for Estonia is positive and converging with European averages.

**2. Universal health coverage: the policy context**

Health policy issues are mostly covered at national level, while municipalities have only a limited role in public health policy through local Health In All Policies and implementation of selected national public health programmes. Currently, the main policy document is the National Health Plan 2009-2020 [3] (NHP).

**3. Monitoring and evaluation for UHC**

The NHP contains measurable targets with specific indicators that are reported annually with outcome reviews every second year. The overarching targets of the NHP are set as life expectancy reaching 75 years for men and 84 years for women by 2020 and healthy life expectancy respectively 60 and 65 years by the same time. While the overarching targets of NHP are measuring health outcomes, other targets focus on access to care, quality of care, health insurance coverage, out-of-pocket payments, financial risk protection, efficiency of resource allocation etc. However, all the other targets are not stratified by gender, region or any socio-economic factor and many important areas (e.g. smoking, alcohol consumption, obesity) are not covered. See Table S1 for all the NHP indicators and their targets.

While the NHP as an overarching health policy was adopted only in 2008, there is a longer tradition in Estonia regarding area specific health strategies [15,16] and institutional development plans [17]. These all have their specialised indicator frameworks for targeting, monitoring and assessing progress made in improving population health. All these specific policy documents feed into the overall health system monitoring and assessment system thus enabling improved governance and policy development. As an example, the EHIF 4-year development plan [17] is the basis for annual health insurance budget and thus provides a frame for UHC and especially for service availability, access, quality and timeliness both in primary and secondary health care.

The monitoring and reporting system of NHP is further supported by health system performance assessment [4] (HSPA) that has been developed based on the example of World Health Report 2000 [18] and international best-practice examples. Accountability for health system performance and therefore it’s assessment for improvement of population health outcomes are commitments of the Tallinn Charter [19] adopted by WHO European Member States in 2008. The main focus of the national HSPA is again on the principles of health service coverage and fair financing that are linked to population health outcomes. See an overview of the HSPA indicator framework in Table S2 (end of file).

Estonia does not have a specific framework for monitoring UHC. However, all the constituent components of the broad view on UHC as defined in the introduction are present in the NHP and HSPA as their indicator frameworks are based on a health system framework [4] (Figure S3) that is closely related to the framework underlying UHC monitoring [20]. The part of the health system framework from Estonian HSPA that covers goals and intermediate goals of health system is used as a base in this paper to assess to what extent the UHC in its entirety is covered there, what is the status of UHC in Estonia and whether any changes are needed to complement the existing policies and their indicator frameworks.


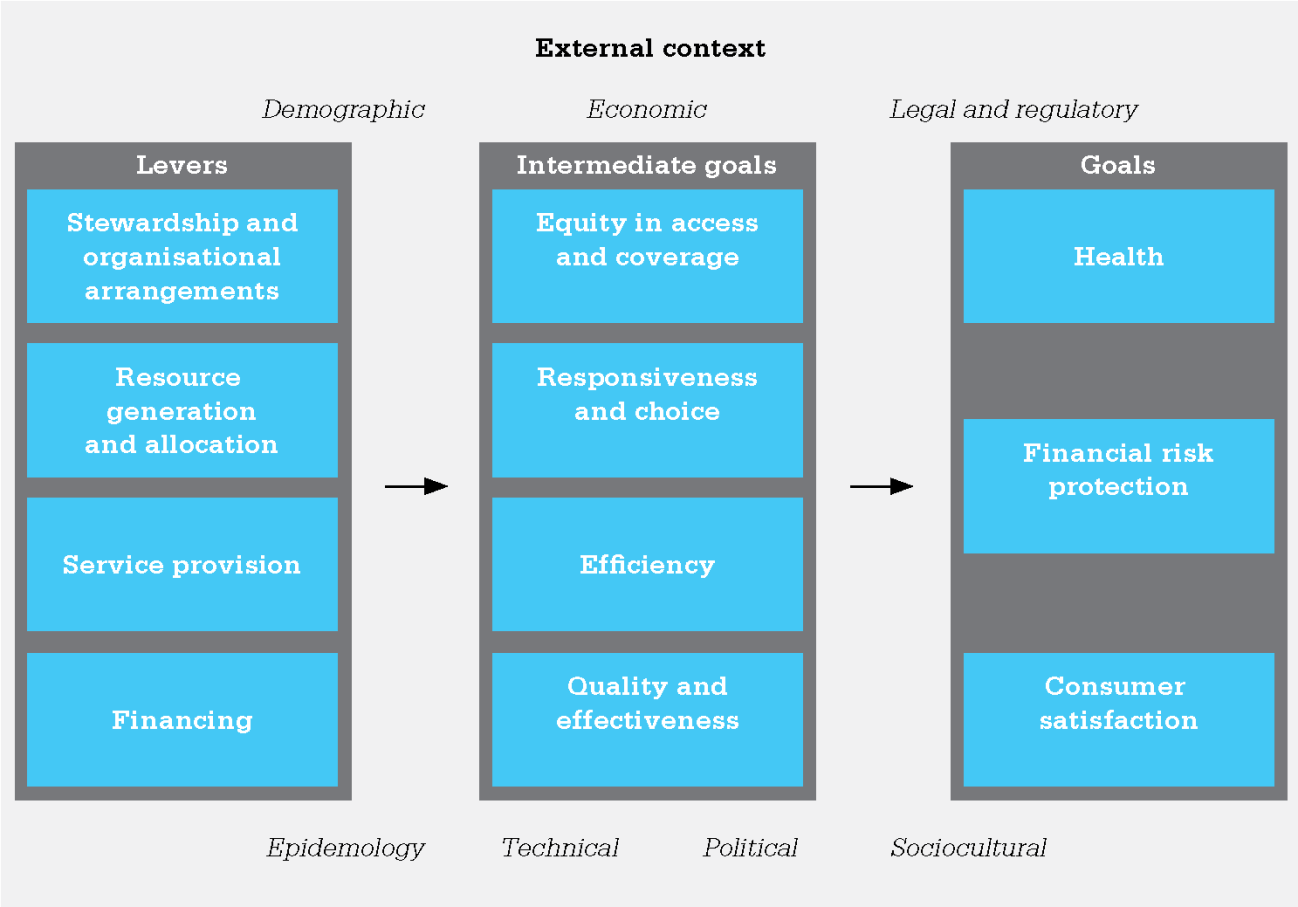


**Figure S3:** Health system framework used in health system performance assessment in Estonia.

Data source: [4]

| **Priority area** | **Indicator** | **Baseline** | **Targeted** | | |
| --- | --- | --- | --- | --- | --- |
|  |  | **2008** | **2012** | **2016** | **2020** |
| **Social cohesion** | Life expectancy at birth, men | 67.36 | 71.2 | 73 | 75 |
|  | Life expectancy at birth, women | 78.45 | 81.1 | 82.5 | 84 |
|  | Disability free life expectancy at birth, men | 49.41 | 56 | 57.5 | 60 |
|  | Disability free life expectancy at birth, women | 55.25 | 60.5 | 62.5 | 65 |
|  | Proportion of population covered with health insurance (%) | 95.2 | 99 | 99 | 100 |
|  | Relative poverty (%) | 18.3 | 16.8 | 16 | 16 |
|  | Poverty risk of children (% of under 15y) | 19.8 | 19 | 18 | 17 |
|  | Longterm unemployment (% of >12m unempl in workforce) | 2.3 | 1.7 | 1.3 | 0.8 |
|  | Suicide mortality | 18.4 | 15 | 12.5 | 10 |
| **Childrens health** | Infant mortality | 4.4 | 3.6 | 3 | 2.6 |
|  | Mortality rate of 0-19y olds | 61 | 46 | 39 | 31 |
|  | Mortality rate of 0-19y olds from accidents and injuries | 30 | 23 | 19 | 15 |
|  | Incidence of mental health problems among 1-19y olds | 2251 | 2058 | 1929 | 1801 |
|  | Proportion of very good assessments of health among 11y, 13y and 15y olds | 31.5 | 32.9 | 33.8 | 34.7 |
| **Environmental health** | Pulmonary mortality | 36.9 | 34.5 | 33 | 31.4 |
|  | Rate of fatal occupational accidents per 100 000 employed persons | 4.5 | 3.6 | 3 | 2.4 |
|  | Number of lost working days from occupational accidents per 100 employed | 20 | 18 | 17 | 15 |
|  | Proportion of people who assess work to be reducing their health | 59 | 50 | 40 | 30 |
|  | Incidence of food-born infectious diseases | 303 | 250 | 200 | 200 |
|  | Proportion of population with access to good quality drinking water | 73 | 86 | 88 | 90 |
|  | Prevalence of people with asthma diagnosis among 16-64y olds | 2.1 | 1.8 | 1.7 | 1.5 |
|  | Annual average concentration of particulate matter (PM10) in outdoor air in Estonian cities (mg/m3) | 20.7 | 18 | 16 | 14 |
| **Health behaviour** | Prevalence of overweight among 16-64y olds | 31 | 28 | 26 | 25 |
|  | Prevalence of obesity among 16-64y olds | 15 | 13 | 13 | 12 |
|  | Proportion of overweight school children | 7.8 | 7 | 6.5 | 6 |
|  | Incidence of HIV | 47.2 | 30 | 20 | 15 |
|  | Proportion of HIIV+ pregnant women | 0.3 | <1 | <1 | <1 |
|  | Proportion of 15-16y olds who have tried illicit drugs | 33.5 | 29 | 24 | 21 |
|  | Mortality of accidents, poisonings and injuries | 121 | 95 | 78 | 61 |
|  | Number of dead from traffic accidents caused by drunk drivers | 53 | 35 | 25 | 15 |
| **Health care** | Number of physicians per 100 000 population | 327 | 320 | 320 | 320 |
|  | Number of nurses per 100 000 population | 656 | 761 | 830 | 900 |
|  | Proportion of population satisfied with health care quality | 69 | 70 | 71 | 72 |
|  | Proportion of population satisfied with access to health care | 60 | 62 | 65 | 68 |
|  | Share of household expenditures (OoP) in total health care expenditure | 24 | <25 | <25 | <25 |

Table S1: Indicators and targets for monitoring and assessing performance of Estonian National Health Plan 2009-2020

Data source: [3]

**4. Progress towards UHC in Estonia**

Financial protection:

Insurance coverage

The main attention of Estonian policy discussions on UHC has been on health insurance coverage of the population which is currently at 95% level [6]. Latter is also among the national health policy indicators with 100% health insurance coverage target by 2020. At the moment, the uninsured are provided emergency care financed from state budget and they are mostly 20 to 60 years old and in majority economically inactive or working abroad [21].

Distribution of OOP

The overall level of out-of-pocket payments (OOP) is again an indicator monitored in the context of the NHP, targeting OOP proportion below 25% in total health care expenditure. While share of OOP was 25% in 2006, it declined to 18% in 2012 [5]. The probable causes of this reduction are manifold including promotion of use of generics in prescription medicine as the latter are the main source of OOP in Estonia [6]. However, the role of economic downturn in reduction of OOP in Estonia is unclear. There are some indications that people may have postponed health care service use (e.g. adults’ dental care that is not included in benefit package) which thus has an impact on health care related household expenditures that are likely highest among poorer households.

While the NHP does not monitor equity of the distribution of OOP, the Estonian Household Budget Survey [22] enables these calculations and there has been some targeted research [23,24] on the topic, also reflected in HSPA reporting. The OOPs for health care services were highest among the poorest households before the economic downturn but the lowest in 2011 as shown on Figure S1. Also, the share of medication expenditure in OOP has declined over the years, especially for high income groups who spend proportionally more on outpatient care while for the poorest quintile medication expenditure is still the biggest source of OOP.

**
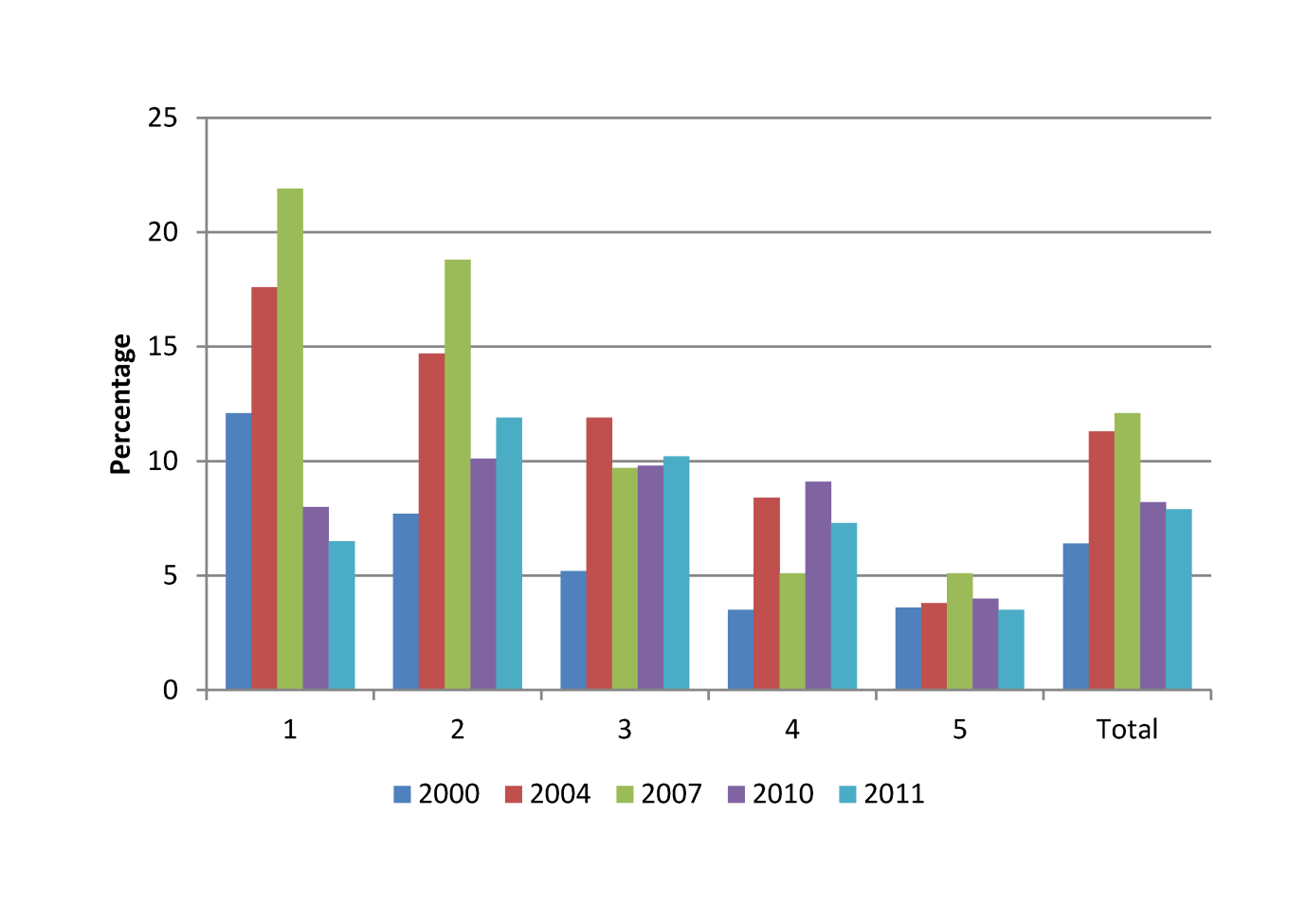
**

**Figure S1:** OOPs as a proportion of total household expenditure by quintiles

Data source: [23]

Catastrophic health expenditures

Household budget data also show that in 2011 the proportion of high health expenditures (>20% of capacity to pay) was lowest among the poorest and richest income quintiles with 6.5% and 3.5% respectively while in the second income quintile this proportion reached 12% (Figure S4). Moreover, the reduction of high health expenditures was most dramatic among the poorest households as only in 2007 almost 22% of these spent more than 20% of their capacity to pay on health care services. Unfortunately, this dataset cannot answer the question whether the need for health care services was met or not. As a result of OOP, about 1% of households in the poorest quintile face poverty in 2011 compared to almost 9% in 2004.


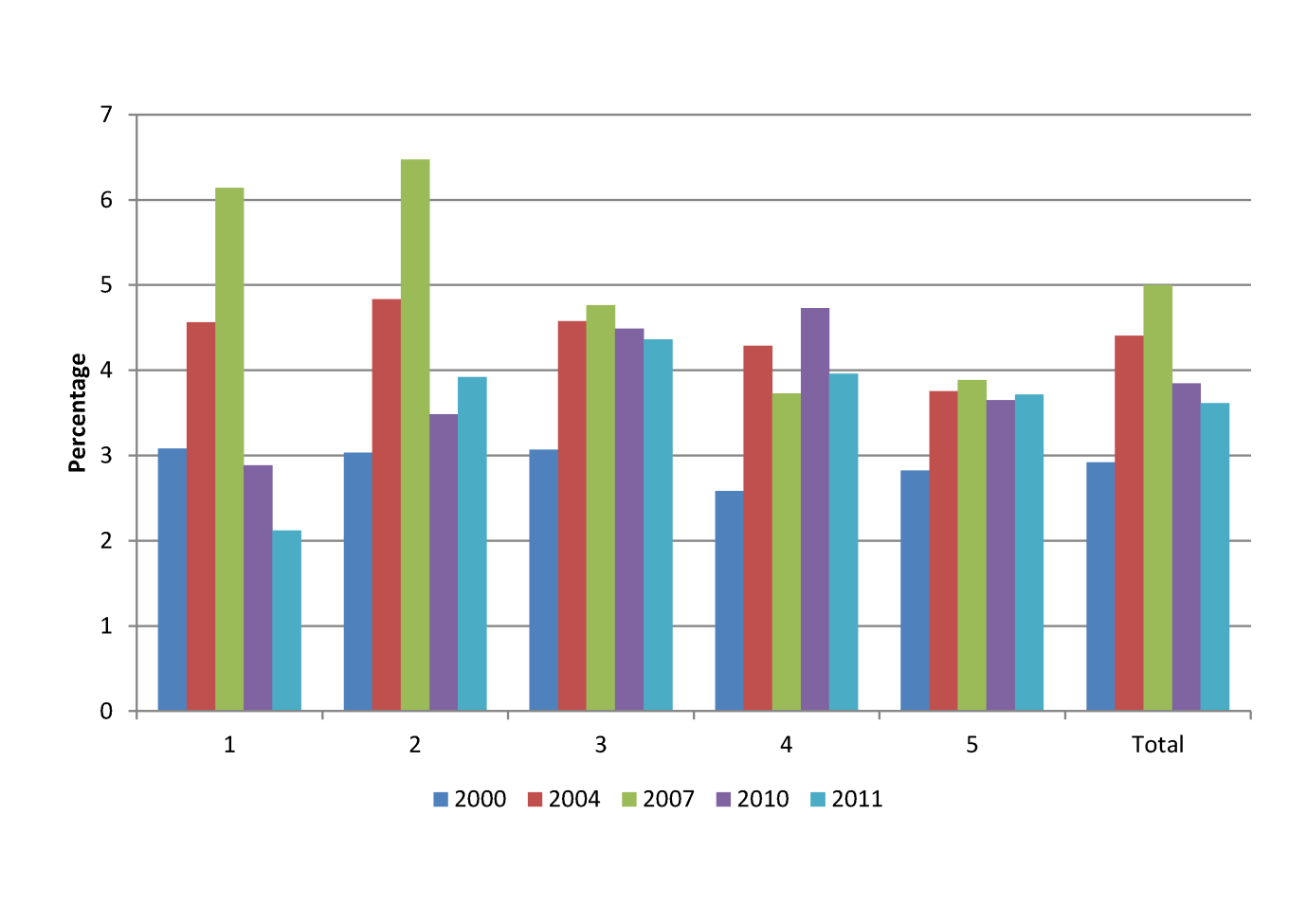


**Figure S4:** Percentage of households with high health payments (> 20% of capacity) by quintile, 2000-2011

Data source: [23]

Responsiveness to need

Access to services

Access to services in Estonia is monitored through population satisfaction surveys, health care utilisation data and by recording waiting time. Out of these, population satisfaction is probably the most frequently used in national discussions. According to the latest population survey 55% of people consider access to health care services in Estonia to be either good or very good [25]. Interestingly, the level of satisfaction with access to care has been stable since 2003 without a clear trend while the overall trend for satisfaction with care quality and the health care system has been positive, even during the economic downturn and 79% of adult population was satisfied with the quality of care in Estonia in 2012 (Figure S5). The NHP also prioritises access to care and sets a target of 68% of population satisfied with access to care by 2020.

For waiting times there are agreed maximum limits which apply to EHIF’s reimbursed care: emergency care should be provided immediately, outpatient specialist care within six weeks and inpatient care within eight months. These waiting time limits are monitored monthly by EHIF and data is published on EHIF webpage [26].


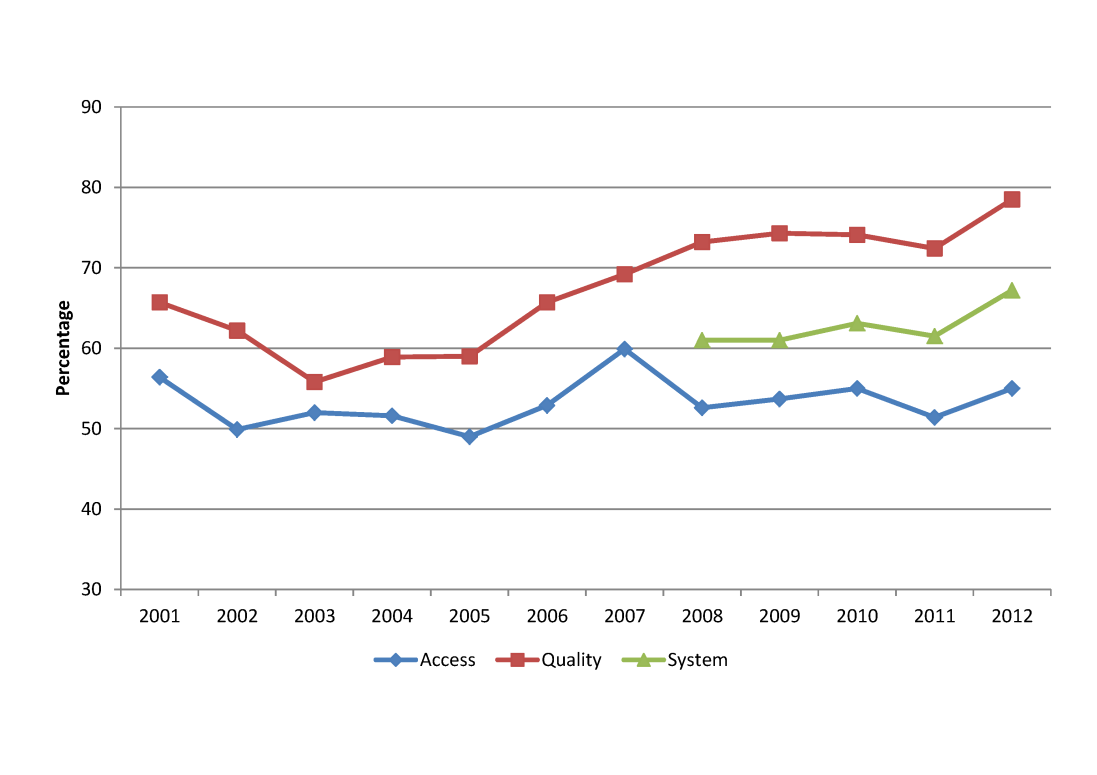


**Figure S5:** Satisfaction with the health system, quality of care and access to care in Estonia 2001-2012 in adult population.

Data source: [25]

Coverage with services

The best example of measurement of coverage with effective services comes from primary health care (PHC). Service coverage in PHC is measured in voluntary Quality Bonus System (QBS) which is a payment scheme for family physicians introduced in 2006 [6]. The QBS includes three service domains: disease prevention, chronic diseases management, and additional activities as surgical and gynaecological procedures at PHC level. There are altogether 45 indicators in these domains with focus on selected disease groups. For an example, effectiveness of hypertension management is measured by several indicators e.g. the share of high risk patients with total cholesterol test done once per year increased from 50% in 2007 to 72% in 2012 with the target at 90%. For diabetes II there are seven indicators e.g. the proportion of high risk patients receiving glycohemoglobin test once per year has increased from 46% in 2007 to 72% in 2012 and for creatinine test from 49% to 74% respectively (target value is 90%).

A key factor in implementing the QBS has been the electronic billing data collection system, which enables monitoring of PHC activities at the patient level without need for additional data collection. As the QBS is a voluntary scheme, it is important that the proportion of family practitioners participating has exceeded 90% by since its introduction.

Allocative and technical efficiency of service provision

The indicators for allocative and technical efficiency are important for identifying possibilities for extending UHC with available resources. The indicators most often used in Estonia in this area are number of hospital beds, average length of stay (ALOS) in hospital and different indicators on physicians and nurses. Volume of hospital beds decreased significantly with hospital network restructuring and by 2011 the number of acute care hospital beds reached 350 per 100 000 population (below EU average) [8]. The ALOS in acute care hospitals decreased in parallel from 17.4 days in 1990 to 5.5 days in 2011 (Figure S6) while the number of physicians per 100 000 population has remained stable (326 in 2011) with nurse-physician ratio remaining at the level of 2 as well. In comparison, the number of nurses per physician is higher in EU on average due to higher availability of nurses.


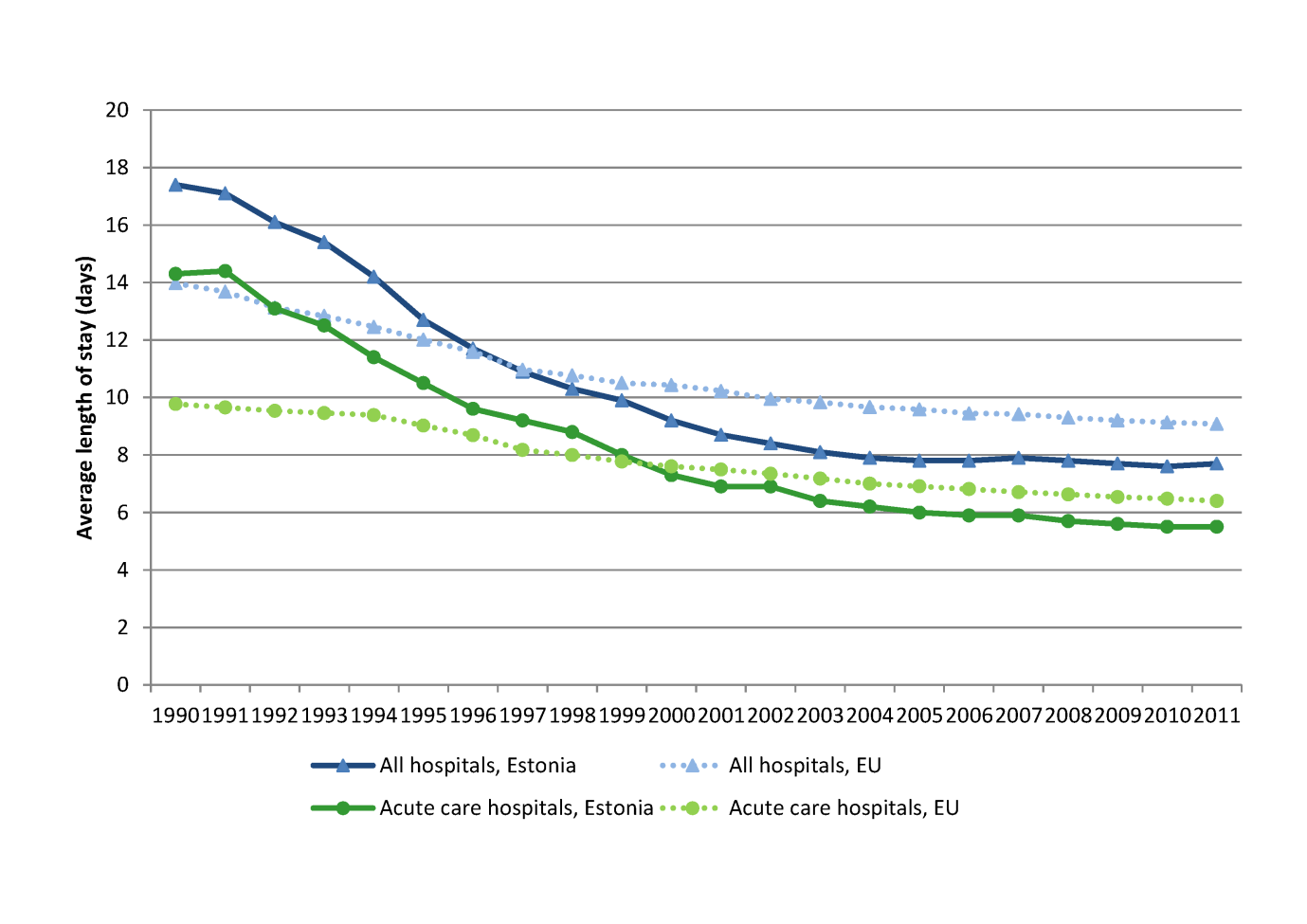


**Figure S6:** Average length of stay (days) in all hospitals and acute hospitals in Estonia and the EU (1990-2011).

Data source: [5]

Quality of services

Measurement of service quality is one of the areas still in development in Estonia. The Estonian Health Insurance Fund (EHIF) started to publish a set of health care quality indicators for acute care hospitals in 2011 [26]. Indicators range from proportion of day surgeries among all surgical interventions to use of specific procedures and rehospitalisation rates for certain diseases. However, on national level, the focus is mostly on patient satisfaction with service quality. As shown on Figure S5, the overall satisfaction with health care quality in adult population reaches 79%. In parallel, 92% of people were satisfied with their last visit to family doctor, 93% with their last hospital visit, 88% with their last outpatient specialist visit in 2012 [25].

Health outcomes

Health status and improvement over time

The leading national indicator for health outcome is life expectancy at birth (LE) as evident from the NHP and its targets. Previous sections of this paper indicated that life expectancy at birth has increased fast since regaining independence in 1991. It is especially noteworthy that LE increased especially fast (about 1 year per annum) during economic downturn [7]. However, LE did not increase from 2011 to 2012 when it was 76.2 compared to 76.3 years in 2011. The main drivers for LE increase from 2000 have been reduction in mortality from cardiovascular disease, injuries and cancers which altogether accounted for 93% of LE improvement during 2000-2008 while diabetes and alcohol related conditions were holding LE increase back [4] (Figure S2).

**
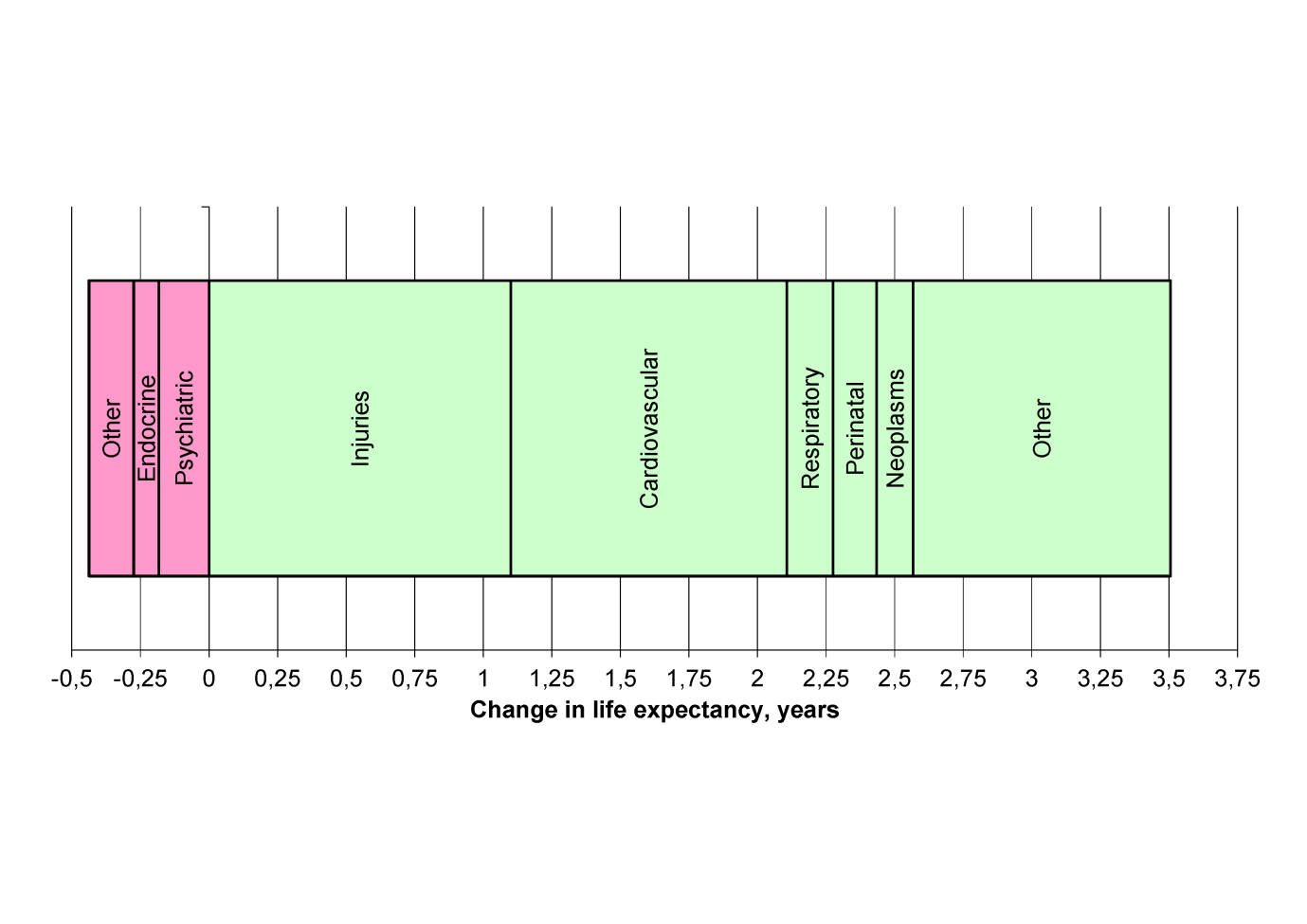
Figure S2:** Life expectancy change in Estonia between 2000-2008 by causes of death.

Data source: [4]

Inequalities in health outcomes

However, there is a significant gender gap in life expectancy of 10 or more years in favour of women already since 1991. There are also regional differences in LE which have declined in recent years and in 2010 only one Estonian county had significantly lower life expectancy than the national best – respectively 72.5 and 77.2 years [6].

More importantly, health outcomes are strongly linked to socioeconomic factors. Data from a population health survey carried out every second year show that since 2004 the proportion of persons assessing their health good or very good is 20 or more percentage points higher among highly educated persons compared to those with lowest education (Figure S7) [27]. This is also the case for income groups that population groups with higher income assess their health as significantly better compared to low income population groups [6]. Indicators and targets in the NHP or other policy documents do not usually use stratification by socio-economic factors.

**
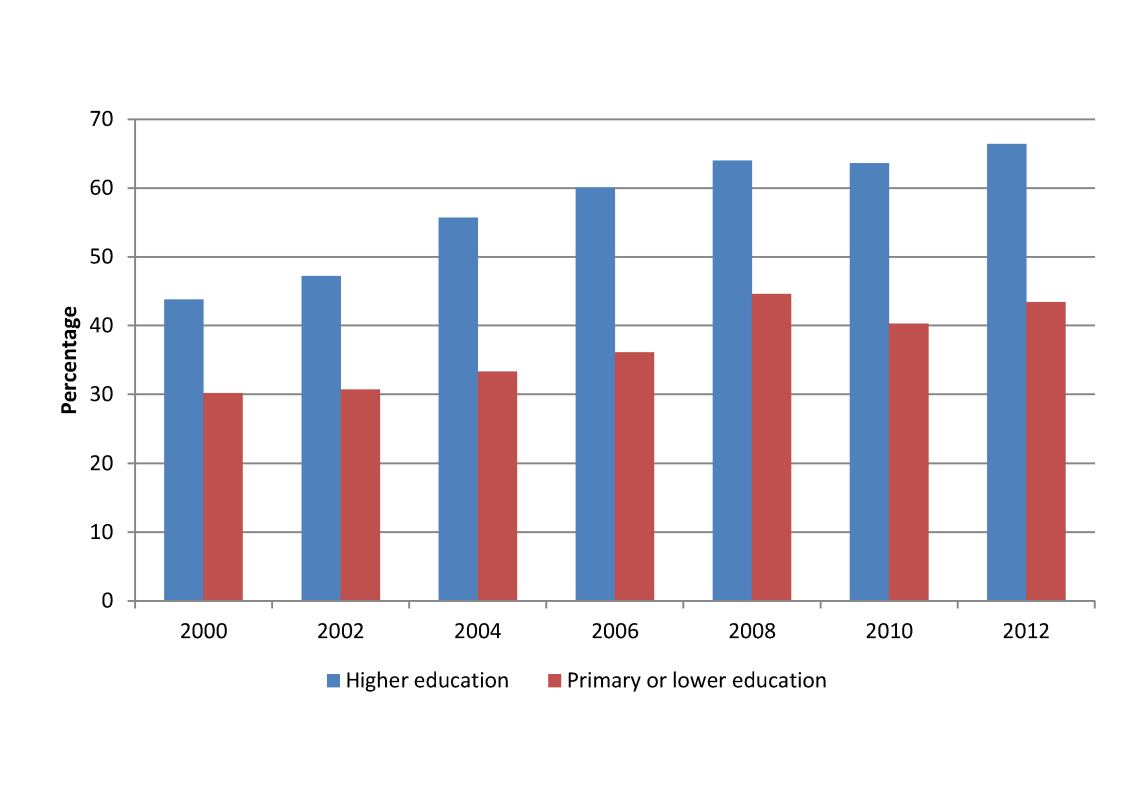
**

**Figure S7:** Proportion of adult population assessing their health as “good” or “reasonably good” by education.

Data source: [27]

**5. Conclusions and recommendations**

The overall health policy framework in Estonia is systematic, comprehensive and built on growing culture of monitoring and performance assessment. The latter was strongly influenced by the World Health Report 2000 which also has a strong link to universal health coverage through the underlying health system frameworks. Thus, the components of UHC can all be found in existing Estonian policy documents, health system performance reporting and their respective monitoring systems. While there is no separate policy, UHC can be monitored and assessed within the existing policy monitoring framework present in Estonia. The backbone of the latter is routine use of different integrated registries and databases, where a person can be tracked through the health system for more than ten years with a wealth of information available on diagnoses, procedures, prescriptions, sick leaves etc.

However, as Estonia lacks a specific framework for UHC monitoring. Even though indicators on main components of UHC can be found in existing monitoring frameworks, development of targeted indicator set would put UHC into stronger policy focus. Moreover, this would help to address UHC issues comprehensively. In comparison, current public debates on UHC tend to focus on health insurance coverage or financial risk protection. Thus, Estonia and its population might benefit from a dedicated indicator set to monitor UHC as a policy objective in its entirety.

There are also data gaps if UHC specific monitoring and assessment framework is considered in Estonia. Most obvious is scarcity of equity aspects in existing indicator frameworks. As an example, the NHP has gender specific targets only for the two main indicators and even then the equity aspect is only implied and not directly addressed while none of the indicators are stratified by socioeconomic, regional or any other social determinants. However, much stronger focus on distribution and equity aspects is needed to assess whether and to what degree the health coverage is truly universal in Estonia and what are the key action points to progress toward UHC.

Further, some component parts of UHC are better covered with indicators than others. As previously already indicated, the field of clinical quality indicators is still very much in development. The initiative in this area is mostly taken by EHIF and also health service providers. As a result, the public discussion and policy development on health care quality until now has been based mostly on population satisfaction indicators while national level information on indicators like 30-day mortality after hospitalisation, level of avoidable hospital admissions and many others is still missing.

Moreover, current data sources in Estonia do not allow measurement of effective coverage. The integrated framework of effective coverage [28] links together true epidemiological need, quality of services, impact of demand and supply on delivery of services while also linking specific services with overall health system and population health outcomes. While there are several population surveys that have a long history and a wealth of self-reported information is collected, there is still little evidence in Estonia about the true epidemiological need in the population due to lack of population health examination studies. A partial solution to that would be use of the national unique personal ID in population health surveys to enable linkage of survey data with existing data sources like health insurance claims and medication prescription databases [21], the population genotyping database [29] or the national central database of electronic medical records [30]. Even though a health examination survey would match self-report with objective measurements in a particular point of time enabling direct association of different factors, linkage of self-report one-to-one with a wealth of background information would also be beneficial to understand status of UHC and coverage of specific health interventions in the country. Additionally, according to the integrated framework of effective coverage, this should at individual level take ex-ante view as the expectation of receiving potential health gain if needed while current view in Estonia seems to more ex-post.

Finally, further focus on developing better linkages between (effective) coverage of specific health interventions or programmes and the health system level health outcomes or coverage would be advisable. This is not only the need in Estonia but also internationally as health policy planners worldwide would very much benefit from better understanding on how to put together a mix of interventions that produces the best possible outcome for the health system as a whole and that also at the best benefit-for-value ratio. An Estonian example here is that national life expectancy is significantly lower than our health care expenditure per capita could produce in comparison to average life expectancy versus health care expenditure relation in OECD countries predicts [31]. It would be important to “decompose” the reasons of this lower than expected return in life expectancy from the available health expenditure for evidence based policy making.

Lessons for other countries from Estonian experience is firstly that comprehensive policy monitoring and assessment system with underlying health system framework enables monitoring of UHC even without a dedicated UHC monitoring framework. This is especially true if extensive routine data sources have been developed and are linked and integrated through comprehensive IT solutions. However, specific focus on UHC and development of dedicated monitoring framework as part of the existing monitoring framework would be one of the key enablers to address existing data gaps and put UHC into strong policy focus for systematic policy development.

| **Box 1: Recommendations based on Estonian experience**  - UHC monitoring should be part of a comprehensive and systematic policy development and monitoring framework that is based on an underlying conceptual health system framework  - Support to system-wide monitoring and performance assessment culture that is linked to policy development processes provides the platform for sustainable UHC improvement  - Development of strong IT platform for routine collection of administrative and other data with possibilities to “track” individuals in the health system over the years, locations and services is important for mainstreaming UHC in the health policy  - Clear definition of UHC as a policy priority accompanied with a clearly defined monitoring framework and institutionalised performance assessment is a key for systematic action |
| --- |

**References**

1. WHO. (2010) The world health report 2010 - Health systems financing: the path to universal coverage. Geneva: World Health Organization.
2. Evans DB, Saksena P, Elovainio R, Boerma T. (2013) Measuring Progress Towards Universal Coverage. Geneva: World Health Organization.
3. Ministry of Social Affairs. (2008) National Health Plan 2009-2020. http://www.sm.ee/fileadmin/meedia/Dokumendid/Tervisevaldkond/Rahvatervis/RTA/ERTA_english.pdf Accessed 2014 Mar. 5.
4. WHO Regional Office for Europe (2010) Estonia, Health System Performance Assessment: 2009 snapshot. Copenhagen: WHO Regional Office for Europe.
5. National Institute for Health Development. (2013) National health accounts. <http://pxweb.tai.ee/esf/pxweb2008/Database_en/HCresources/10Expenditure/10Expenditure.asp> Accessed 2014 Mar. 5.
6. Lai T, Habicht T, Kahur K, Kiivet RA, Reinap M, et al. (2013) Estonia: health system review. *Health Systems in Transition.* Copenhagen: WHO Office for European Region.
7. Statistics Estonia. (2014) Life expectancy by sex and age. http://pub.stat.ee/px-web.2001/Dialog/varval.asp?ma=PO045&ti=LIFE+EXPECTANCY+BY+SEX+AND+AGE&path=../I_Databas/Population/01Population_indicators_and_composition/02Main_demographic_indicators/&lang=1 Accessed 2014 Mar. 5.
8. WHO Regional Office for Europe. (2014) European Health for All Database (HFA-DB). http://data.euro.who.int/hfadb Accessed 2014 Mar. 5.
9. Estonian Institute of Economic Research. (2013) Alcohol market, consumption and harms in Estonia: Yearbook 2013. https://intra.tai.ee/images/prints/documents/138304634141_Alkoholi_aastaraamat_2013.pdf Accessed 2014 Mar. 5.
10. Tekkel M, Veidemann T. (2013) Health behaviour among Estonian adult population, 2012. Tallinn: National Institute for Health Development.
11. Lai T, Köhler K. (2009) Burden of disease of Estonian population. http://www.sm.ee/fileadmin/meedia/Dokumendid/V2ljaanded/Toimetised/2009/series_20091eng.pdf Accessed 2014 Mar. 5.
12. Lai T, Habicht J, Kiivet RA. (2009) Measuring burden of disease in Estonia to support public health policy. Eur J Public Health 19: 541-547.
13. Bank of Estonia. (2014) Annual indicators of Estonian economy. http://statistika.eestipank.ee/?lng=et#listMenu/1017/treeMenu/MAJANDUSKOOND Accessed 2014 Mar. 5.
14. European Commission. (2014) Eurostat statistical database. http://epp.eurostat.ec.europa.eu/portal/page/portal/statistics/search_database Accessed 2014 Mar. 5.
15. Ministry of Social Affairs. (2005) Estonian National Strategy for Prevention of Cardiovascular Diseases 2005-2020. Tallinn: Ministry of Social Affairs.
16. Ministry of Social Affairs. (2007) National Cancer Strategy 2007-2015. http://www2.tai.ee/teated/arenduskeskus/Vahistrateegia/National_Cancer_strategy_2007_2015eng.pdf Accessed 2014 Mar. 5.
17. Estonian Health Insurance Fund. (2012) Eesti Haigekassa arengukava 2012-2015. [Strategic Development Plan of Estonian Health Insurance Fund 2012-2015]. http://www.haigekassa.ee/uploads/userfiles/Otsus_nr23_arengukava_2012_2015.pdf Accessed 2014 Mar. 5.
18. WHO. (2000) The world health report 2000 - Health systems: improving performance. Geneva: World Health Organization.
19. WHO Regional Office for Europe. (2008) The Tallinn Charter: Health Systems for Health and Wealth. Copenhagen: World Health Organization.
20. WHO. (2013) Measurement and monitoring of Universal Health Coverage: Development of guidance through country case studies. Geneva: WHO.
21. Estonian Health Insurance Fund. (2013) Estonian Health Insurance Fund. http://www.haigekassa.ee/eng/ Accessed 2014 Mar. 5.
22. Statistics Estonia. (2013) Household Budget Survey (HBS). Tallinn: Statistics Estonia.
23. Võrk A. (In-press) Out-of-pocket payment and catastrophic expenditure in Estonia 2000-2011. Copenhagen, WHO Regional Office for Europe.
24. Võrk A, Saluse J, Habicht J. (2009) Income-related inequality in health care financing and utilization in Estonia 2000-2007. http://ee.euro.who.int/E92592.pdf Accessed 2014 Mar. 5.
25. Ministry of Social Affairs, Estonian Health Insurance Fund. (2013) Elanike hinnangud tervisele ja arstiabile 2012. [Population satisfaction with health and healthcare 2012]. http://www.haigekassa.ee/uploads/userfiles/Elanike%20hinnangud%20tervisele%20ja%20arstiabile_2012_raport_GfK.pdf Accessed 2014 Mar. 5.
26. Estonian Health Insurance Fund. (2014) Eriarstiabi järjekorrad. [Waiting times for providers of specialised care]. http://www.haigekassa.ee/kindlustatule/jarjekorrad/eriarstiabi Accessed 2014 Mar. 5.
27. National Institute for Health Development. (2013) Self-estimated health by gender and education. Tallinn: National Institute for Health Development.
28. Shengelia B, Tandon A, Adams OB, Murray CJ. (2005) Access, utilization, quality, and effective coverage: an integrated conceptual framework and measurement strategy. Soc Sci Med 61: 97-109.
29. Estonian Genome Center of University of Tartu. (2014) Estonian Genome Center of University of Tartu. http://www.geenivaramu.ee/en/ Accessed 2014 Mar. 5.
30. Estonian e-Health Foundation. (2014) Estonian e-Health Foundation. http://e-tervis.ee/index.php/en/ Accessed 2014 Mar. 5.
31. OECD. (2013) OECD Health Data 2013. http://www.oecd.org/health/health-systems/oecdhealthdata.htm Accessed 2014 Mar. 5.

| **Performance dimension** | **Performance subdimension (if any)** | **Policy questions addressed** | **Performance indicators** |
| --- | --- | --- | --- |
| Health status (level and distribution) | Life expectancy | How healthy are Estonians? | Change in life expectancy at birth in Estonia, by sex |
|  |  |  | Male and female life expectancy at birth in selected European countries |
|  |  |  | Changes in life expectancy 2000 versus 2008 attributable to different disease groups |
|  | Life expectancy without disabilities | How many years do Estonians live without disability? | Disability-free life expectancy |
|  | Self-assessed health | How do Estonians assess their health? | Self assessed health |
|  | Changes in mortality rates | What are the main challenges and opportunities for improvement? | Infant mortality rate |
|  |  |  | Child mortality rate |
|  |  |  | Avoidable mortality |
|  |  |  | Potential gains in life expectancy in days if avoidable mortality were avoided in 2008 |
|  | Burden of disease | How many life-years do Estonians lose due to premature mortality and ill health? | Main disease groups causing the burden of disease |
|  |  |  | Regional levels of the burden of disease (disability-adjusted life-years per 1000 persons) |
| Health behaviour and health promotion |  | How well is the health system performing in keeping people healthy? | Immunization rates of two-year-olds with national immunization calendar vaccines |
|  |  |  | Proportion of daily smokers aged 15+ years |
|  |  |  | Consumption of pure alcohol per person |
|  |  |  | Overweight |
|  |  |  | Prevalence of overweight and obesity |
|  |  |  | Physical activity |
| Broader determinants of health | Social determinants of health | How is Estonia’s health system doing with regards to broader social determinants of health? | Level of education |
|  |  |  | Unemployment rate |
|  | Environmental health | Are there environmental health issues that threaten the health of Estonians? Can they be reduced? | Percentage of population having access to clean drinking-water |
|  |  |  | Average concentration of small particles in the air in cities |
|  | Occupational health | Are there occupational health issues threatening the health of Estonians and can they be reduced? | Incidence of occupational diseases |
|  |  |  | Deaths from work-related accidents |
| Responsiveness of the health system |  | How responsive is Estonia’s health system in terms of respect for people and a client orientation? | Satisfaction with the health care |
|  |  |  | Satisfaction with the quality of health care services |
|  |  |  | Satisfaction with access to health care services |
|  |  |  | Satisfaction with hospital care and primary care during the last visit |
|  |  |  | Satisfaction with health care benefit package |
| Fair financing, financial protection and coverage | Health system expenditures | Is the level of health expenditure sufficient and sustainable? | Government spending on health compared with overall government spending |
|  |  |  | Out-of-pocket and other private expenditure on health as a percentage of total health expenditure |
|  |  |  | Government spending on health as a percentage of gross domestic product (GDP) |
|  |  |  | Out-of-pocket payments as a percentage of GDP per capita |
|  | Fairness of health system financing in Estonia | Is the burden of financing for health borne proportionally more by the wealthier people in Estonia? | Sources of health care financing as a percentage of total health care expenditures. |
|  | Protection against the financial risk of ill health | Is the health system protecting the poorest people against the financial risk of ill health? | Total household out-of-pocket payments and as a percentage of total household expenditure by income quintile |
|  |  |  | Proportion of households impoverished due to out-of-pocket payments |
|  | Health system coverage | Do Estonians have universal health care coverage? | Total population health service coverage (no figure) |
| Efficiency and effectiveness of the health system | Technical efficiency | Is Estonia’s health system technically efficient? | Hospital beds per 1 000 000 population |
|  |  |  | Average length of stay, all hospitals |
|  |  |  | Bed occupancy rate (%), acute care hospitals only |
|  |  |  | Physicians per 100 hospital beds |
|  | Allocative efficiency | Are health system resources allocated in a way that promotes better health system value? | Primary health care and inpatient expenditure compared with total health system expenditure |
|  |  |  | GPs and specialist physicians per 100 000 population |
|  |  |  | Ratio of nurses to physicians |
|  |  |  | GP utilization versus hospitalization rate |
|  | Overall health system efficiency | Is the health system effective in using its resources? | No indicator available |
| Access to health care services | Utilization of health care services | Are those needing care able to access services when they need them? | Rates of inpatient admissions and outpatient contacts |
|  |  |  | Average hospital waiting times for inpatient, outpatient and ambulatory care |
|  |  |  | Distribution of waiting times for hospital inpatient care in large Estonian hospitals as of July 1, 2009 |
|  |  |  | Reported waiting times for specialist services |
|  |  |  | Reported waiting times for access to General Practitioners |
|  | Equity in utilization of health care services | Does the system provide health care equitably to all residents of the country? | Percentage of population reporting problems accessing dental care by income quintile |
|  |  |  | Relationships between standardized mortality rates compared with hospitalization rates |
|  |  |  | Relationships between standardized mortality rates compared with GP contacts |
| Quality and safety of health care services |  | During their interaction with the health care, are patients receiving care of sufficiently high quality? | Hospital readmission rates for acute myocardial infarction and for asthma |

Table S2: Indicator areas used in national health system performance assessment (HSPA)

Data Source: [9]
